# Investigating the Sensitivity of the Diffusion MRI Signal to Magnetization Transfer and Permeability via Monte‐Carlo Simulations

**DOI:** 10.1002/mrm.70378

**Published:** 2026-04-12

**Authors:** Zhiyu Zheng, Karla L. Miller, Benjamin C. Tendler, Michiel Cottaar

**Affiliations:** ^1^ Oxford Centre for Integrative Neuroimaging (OxCIN), FMRIB, Nuffield Department of Clinical Neurosciences, University of Oxford Oxford Oxfordshire UK

**Keywords:** diffusion modeling, exchange, magnetization transfer, microstructure, Monte‐Carlo simulation

## Abstract

**Purpose:**

Magnetization transfer (MT) and water exchange via permeability operate on a similar spatiotemporal scale to water diffusion. In this study, we use a simulation‐based approach to characterize how MT and permeability impact (1) diffusion‐weighted MRI (dMRI) measurements from cylindrical substrates and (2) parameter estimation using a two‐compartment model of white matter.

**Methods:**

We used Monte‐Carlo simulations to model the dMRI signal inside and outside axons by simulating signals from parallel cylinders with different diameters and volume densities. We subsequently introduced membrane permeability and MT at the cylinder walls to investigate their impact on the dMRI signal. We fitted a two‐compartment model to the simulated signal to produce estimates of the cylinder diameter and density. We evaluated the impact of MT and permeability by comparing the fitted diameter and density to the simulated ground truth.

**Results:**

Permeability leads to underestimation (up to 100%) of cylinder diameter and density. Specifically, by enabling isochromats to escape from restrictions and diffuse more freely, permeability makes the overall displacement profile closer to the extra‐axonal displacement profile. MT had limited effects on diameter estimation but caused substantial bias (20%–50%) in volume density estimates depending on the ratio of the intra‐axonal and extra‐axonal volume fraction. This is due to the intra‐axonal and extra‐axonal space having different surface‐to‐volume ratios and therefore different surface relaxation rates.

**Conclusion:**

Permeability and MT can considerably influence the dMRI signal. They increase the relative contribution from larger cylinders to the dMRI signal and bias microstructural parameter estimates derived from dMRI data.

## 1 Introduction

Diffusion‐weighted MRI (dMRI) is a leading technique for the noninvasive characterization of tissue microstructure. While MRI voxels are typically on the order of a cubic millimeter, dMRI yields image contrast driven by the diffusion of water in tissue on the order of micrometers. Measured signals are sensitive to changes in cellular morphology and density, providing a basis to characterize brain microstructure, composition, and pathological changes.

To estimate cellular properties from dMRI, microstructure models are fit to experimental data acquired in multiple diffusion regimes (e.g., with different gradient orientations/durations/amplitudes/waveforms or diffusion times). Typically, these models condense a complicated tissue environment into a few distinct compartments corresponding to key tissue features. For example, a widely used signal model for characterizing white matter is AxCaliber [1, 2, 3]. AxCaliber is the simplest dMRI model that can provide estimates of two tissue properties that have attracted considerable research interest: axon diameter and axon density (volume fraction). It models the dMRI signal as two compartments corresponding to intra‐axonal and extra‐axonal water. The intra‐axonal compartment is modeled as parallel cylinders, which enables estimation of axon diameter. This is important as many other common dMRI models model intra‐axonal compartment as zero‐diameter sticks (e.g., NODDI [4], Standard Model [5]) and thus don't provide diameter estimates. The extra‐axonal compartment is modeled as an isotropic Gaussian diffusion tensor. AxCaliber does not include features commonly included in other models, like fiber dispersion or contributions from free water (e.g., ActiveAx [6, 7]).

Axon diameter has been reported to correlate with neuron's conduction velocity [8, 9] and myelination level [10]. Recently, axon diameter mapping has also been used to study the development of the human nervous system noninvasively [11, 12]. Axon density is also closely linked to neurodegenerative diseases as it can (indirectly) reflect pathological cell loss or neuron regeneration [13, 14, 15]. In addition, axon diameter is hard to estimate using clinical scanners and even advanced research scanners as most axon diameters are below the resolution limit set by the gradient strength [16, 17] and the estimation tends to be biased toward the larger axons [18].

A common feature of AxCaliber and other multi‐compartment dMRI signal models is the assumption that signal attenuation (diffusion‐weighted signals relative to *b* = 0) is driven solely by diffusion effects. However, several other effects occur at similar spatiotemporal scales to diffusion, including water exchange across semipermeable membranes [19] and magnetization transfer (MT) [20]. Membrane permeability arises from water passing through cross‐membrane transport proteins (e.g., aquaporins) and/or osmosis [21]. It allows magnetization carried by water molecules and other particles to enter and escape from compartments, leading to a further change in displacement profiles and signal amplitude beyond diffusion‐related changes [19, 22, 23, 24, 25]. MT describes the transfer of magnetization between different molecules. MT is of most interest when magnetization transfers between a free water molecule and a macromolecule, the latter being characterized by very short transverse relaxation times (< 1 ms [26, 27]). Magnetization that has transferred from water molecules into a macromolecule will typically vanish before it can be measured with MRI, reducing the signal amplitude. Since most current models attribute all signal attenuation to diffusion, MT and permeability‐induced signal attenuation is a potential source of bias to fitted model parameters. For example, these effects could contribute to the bias observed between axon diameter estimates from dMRI and histology‐derived estimates [28, 29, 30, 31, 32, 33, 34, 35, 36].

To date, while there are established experimental methods to investigate permeability in tissue using MRI [37, 38, 39, 40, 41], parameter estimation is typically performed using analytical models consisting of multiple Gaussian compartments. These models do not incorporate time‐dependent diffusion properties arising from geometries of restrictions (preventing the estimation of axon diameter) and cannot be easily translated to more realistic tissue models [24, 42, 43] or geometries. As for MT, no analytical solutions exist when considering dMRI signals.

One approach to investigate the impact of permeability and MT on the dMRI signal is via Monte‐Carlo simulations, which can incorporate tissue models of arbitrary complexity. In this project, we use Monte‐Carlo simulations to investigate how the estimation of microstructural tissue features in models of white matter from dMRI are impacted by the contribution of permeability and MT. We used a novel Monte‐Carlo simulator developed by our research group, MCMRSimulator [44], that incorporates the effects of diffusion, permeability, MT, and off‐resonance field perturbations. We have previously used this simulator to demonstrate that permeability and MT can affect size estimations in porous media with parallel plate geometry [45].

More specifically, in this work we simulated the dMRI signal generated by isochromats (i.e., an ensemble of spins experiencing the same off‐resonance field) in substrates consisting of randomly distributed parallel cylinders. Parallel cylinders is a commonly used approximation for axons in white matter and our simulations included both fixed and distributed diameters and incorporated different levels of permeability and MT. We subsequently fit a two‐compartment (intra‐axonal and extra‐axonal) dMRI model to the signal to estimate the axon diameter and density and evaluated the parametric estimation bias introduced by permeability and MT. Like most dMRI models, this model does not consider the effects of permeability and MT, which allows us to evaluate the parametric estimation bias introduced by ignoring these effects in the model fitting.

## 2 Methods

### 2.1 Monte‐Carlo Simulation

All simulations were performed using MCMRSimulator v0.9 [44], a Julia language package developed for modeling the impact of diffusion, relaxation, MT, permeability and off‐resonance on different MRI sequences and microstructural substrates. MCMRSimulator has been comprehensively validated against analytical models by Cottaar et al. [44]. Below we describe the implementation of permeability and MT with MCMRSimulator, and the subsequent simulation study.

#### 2.1.1 General Simulation Parameters

##### 2.1.1.1 Substrate

**FIGURE 1:**
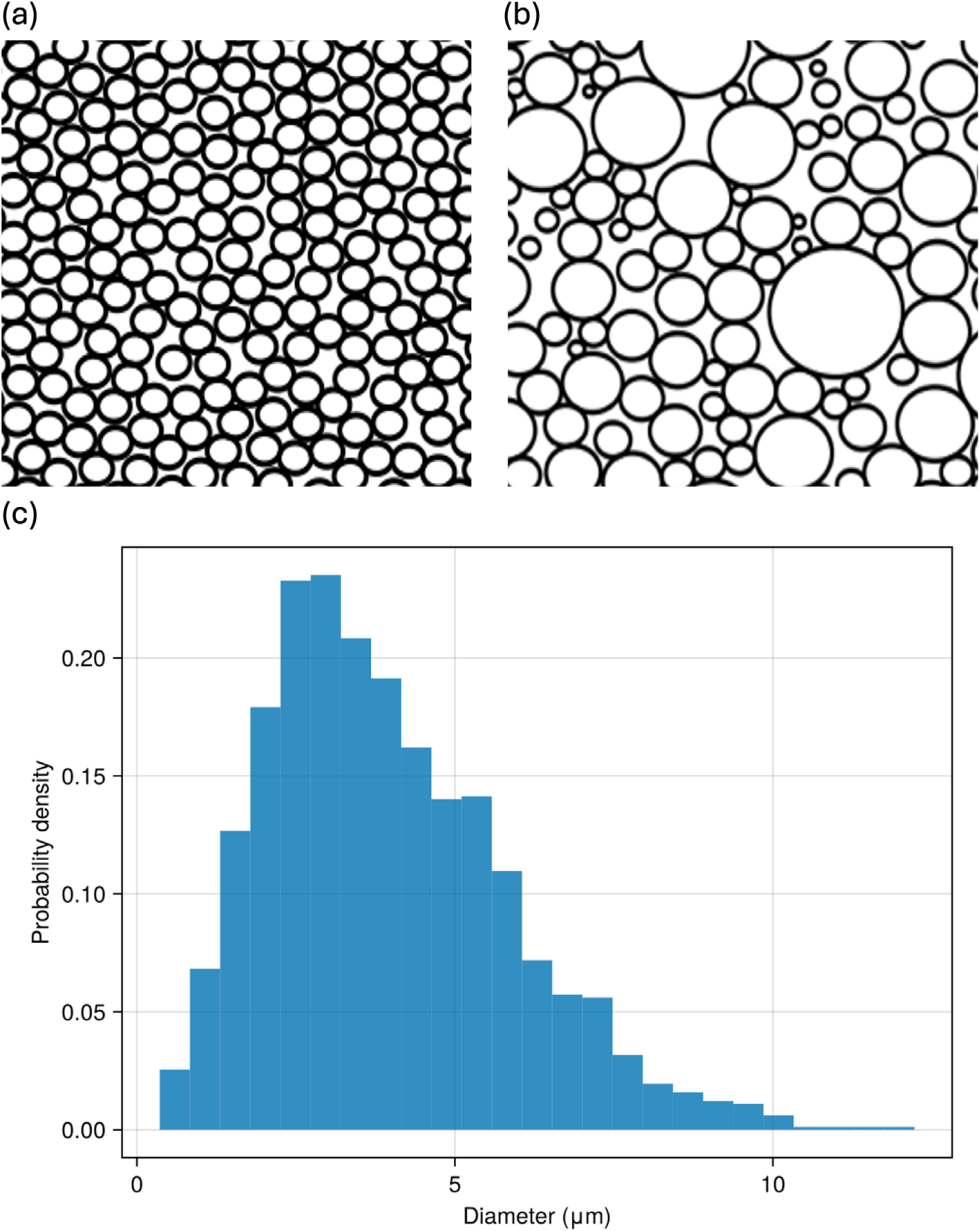
Investigated Monte‐Carlo substrates. (a, b) Simulated substrates consisting of (1) randomly distributed cylinders with an identical diameter (a) and (2) randomly distributed cylinders with Gamma distributed diameters (b). The Gamma distribution corresponds to a mean of 4 μm and standard deviation of 1 μm^2^ (c). Default simulations were performed with a fixed intra‐cylinder volume fraction of 0.65.

All experiments simulated isochromats diffusing inside randomly distributed parallel cylinders and the free space outside the cylinders. Figure 1 shows two example substrates with (a) uniform and (b) distributed diameters. The cylinder walls were approximated as having zero thickness as tissue boundaries with finite thickness have not been implemented in the MCMRSimulator. Finite thickness boundaries wouldn't substantially change the results we demonstrate below but will create complex simulation issues in isochromat's initialization, movement trajectory across the boundary and permeability control. Two sets of simulations were performed: (1) all cylinders have a uniform diameter, with the diameter varying from 0.6 to 11 μm across different substrates (fixed diameter case); (2) cylinders have a distribution of diameters corresponding to a Gamma distribution with a mean of 4 μm and variance of 1 μm^2^ (distributed diameter case). These diameter range and distributions were chosen based on previous microscopy and MRI studies [29, 36, 46, 47]. For these simulations, the volume fraction inside the cylinders was set to 0.65 to approximate realistic axon densities in white matter [48, 49, 50, 51]. Cylinder packing was performed by randomly distributing the number of cylinders needed to achieve the given density and then using distance‐dependent repulsion between cylinders to eliminate overlaps among them to create a near‐homogeneous spatial distribution of cylinders.

##### 2.1.1.2 Sequence Parameters

We used the original AxCaliber pulsed gradient spin echo sequence parameters for our simulations [1]. Specifically, we adopt a gradient duration (*δ*) of 2.5 ms; gradient strengths incremented from 0 to 1200 mT/m with steps of 80 mT/m; diffusion times (Δ) of {10, 15, 20, 30, 40, 50, 60, 80} ms; TE of 166 ms. The diffusion‐weighting gradients were applied perpendicular to the principal axis of the cylinder. These gradient strengths can only be achieved with specialized preclinical systems but they attenuate the gradient resolution limit [16, 17] of dMRI for improved axon diameter estimation.

##### 2.1.1.3 Monte‐Carlo Simulation Setup

Even within a 1 mm^3^ voxel there are more than 10^20^ spins which are computationally unrealistic to simulate. Although no two spins will undergo identical random motion, their net magnetization can be represented by a random subset of trajectories and spin histories [52]. Therefore, each simulated trajectory represents many physical spins, which allows the associated magnetization to be modeled as a classical system, that is, an isochromat.

250 000 isochromats were simulated in each experiment to achieve a good balance of accuracy and simulation speed. The isochromats were initially randomly distributed over an isotropic voxel with its side length equal to 100 times the mean cylinder diameter. For all isochromats, the intrinsic diffusivity was set to 2.3 μm^2^/ms to approximate the intra‐axonal axial diffusivity previously reported in human tissue [53]. The intrinsic relaxation times (*T*_1_, *T*_2_) affect all isochromats equally irrespective of their diffusion profile, leading to the same impact on the diffusion and nondiffusion weighted signal. Therefore, they have no effect on the resulting axon density and diameter, which are estimated from the diffusion attenuation (i.e., ratio of the diffusion and nondiffusion‐weighted signal). Here, we arbitrarily set these intrinsic relaxation times to infinity. The incorporation of MT introduces an effective *T*_1_ and, more importantly for this work, a *T*_2_ difference between compartments, which we will discuss in Section 2.1.2.2.

#### 2.1.2 Implementation of MT and Permeability

##### 2.1.2.1 Permeability

**FIGURE 2:**
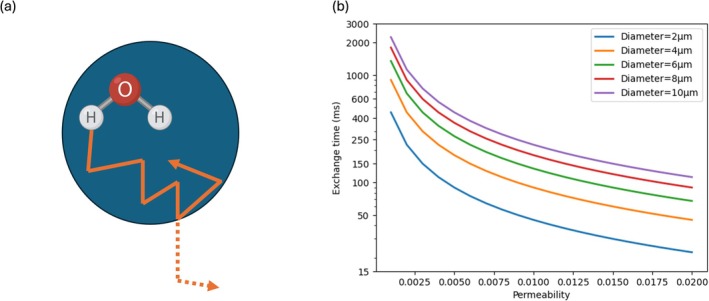
Permeability implementation: (a) If a water molecule collides with an obstruction (cylinder wall), it has a probability of continuing in the original direction and crossing the membrane (dashed line), rather than being reflected (solid line). The probability of crossing is defined as the *permeability* parameter in the simulation. (b) Mapping between *permeability* and exchange time at different cylinder diameters. Note that the exchange time depends on the diameter because opportunities for isochromats to exchange are driven in part by surface‐to‐volume ratio.

To model permeability, we introduced a geometry‐independent parameter to characterize the probability of an isochromat passing through an obstruction for a simulation with a 1 ms timestep. As shown in Figure 2a, the isochromat keeps its original direction of travel if it passes through the obstruction (dashed line) and gets elastically reflected otherwise (solid line). The permeability parameter sets the local probability of isochromats passing the membrane and indirectly controls the exchange time. It was varied across a wide range (0.001–0.02) to explore different membrane types and different membrane states.

A common parameter to characterize membrane permeability is exchange time [18, 24, 25, 54, 55]. It is an exponential decay coefficient characterizing the average time it takes for a particle in one compartment to exchange to another compartment. The exchange process can be described by the following exponential relationship:

(1) $$N_{\text{exchanged}}=\left(1-e^{-\frac{t}{\tau }}\right)N_{\text{initial}},$$

where *τ* is the exchange time constant, *N*_exchanged_ is the number of particles that have exchanged to another compartment, and *N*_initial_ is the number of particles initially inside the compartment.

The exchange time can be analytically derived from the rate of isochromats passing the surface, with previous work [44] defining the exchange rate *R*:

(2) $$R=\text{permeability}\cdot \sqrt{\frac{D}{\pi }}\cdot \frac{S}{V},$$

where permeability is the probability of an isochromat passing through an obstruction, *D* is the intrinsic diffusivity, *S* is the surface area between the two exchanging compartments and *V* is the total (intra‐axonal and extra‐axonal) volume. *S*/*V* is the surface‐to‐volume ratio and for cylinders:

(3) $$\frac{S}{V}=\frac{2S_{\mathrm{cyl}}}{V_{i}}\cdot \frac{V_{i}}{V}=2\cdot \frac{2\pi \mathrm{rl}}{\pi r^{2}l}f_{i}=2\cdot \frac{2f_{i}}{r}=\frac{4f_{i}}{r},$$

where *S*_cyl_ is the surface area of the cylinder, *V*_*i*_ is the intra‐axonal volume, *f*_*i*_ is the intra‐axonal volume fraction, *r* is the radius, *l* is the cylinder length. The factor “2” in front of *S*_cyl_ accounts for exchange from the intra‐ to the extra‐axonal space and the other way around, both of which occur across the cylinder surfaces with the same surface area *S*_cyl_. As the exchange time constant *τ* is the reciprocal of exchange rate *R*, we can combine Equation (2) and Equation (3) to have

(4) $$\tau =\frac{1}{R}=\frac{r}{4f_{i}\cdot \text{permeability}}\sqrt{\frac{\pi }{D}}$$

For a cylinder diameter of 2 μm, our chosen range of permeabilities corresponds to exchange times of 25–508 ms. As shown in Figure 2b, cylinder diameters are closely related to the exchange time. We describe how this relationship impacts parameter estimation in Section 4.1.

##### 2.1.2.2 MT

###### 2.1.2.2.1 Modeling Approach

**FIGURE 3:**
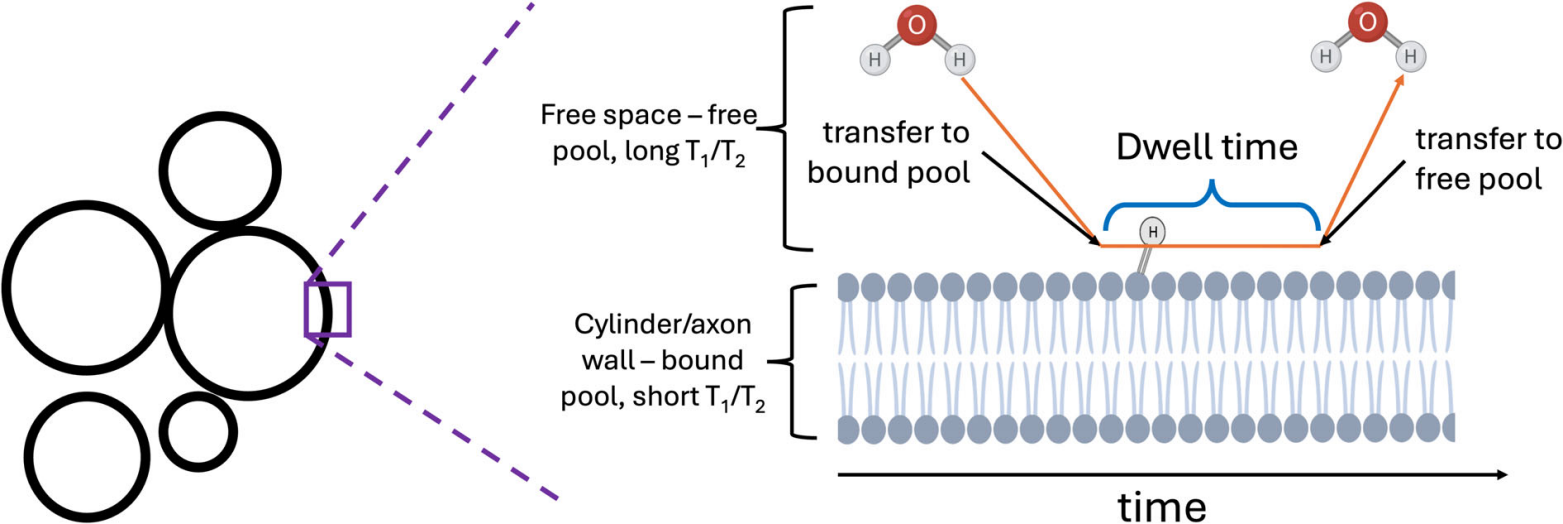
Bound pool interaction mechanism at the cylinder wall: Isochromats are split into a free pool and bound pool. The bound pool is localized at the obstructions (membrane in this case). When an isochromat in the free pool encounters the membrane, there is a fixed probability that it will transfer into the bound pool and get spatially attached to the membrane. This is controlled by both surface density and dwell time. The dwell time is the characteristic timescale for a bounded isochromat to be transferred back into the free pool. Note that this interaction happens on both sides of the axon wall even though only one side is shown in this illustration.

We model MT as an exchange process at the membrane. Specifically, MT occurs when an isochromat collides with an obstruction (e.g., a cylinder wall). To model the interaction during the collision, a bound‐pool interaction model was implemented. As shown in Figure 3, isochromats are divided into two groups of protons that exchange with each other—the bound pool and the free pool. The bound pool represents ensembles of protons on macromolecules localized at user‐defined obstructions and has a very short transverse relaxation time, *T*_2_. The free pool represents ensembles of protons in water molecules and occupies the remaining space. When an isochromat encounters an obstruction, it has a certain probability of getting bound to the obstruction (i.e., transferred into the bound pool). Once bound, it will experience very short *T*_2_ and quickly loses all its transverse magnetization. After some time, it is released from the bound pool and subsequently takes a new random step which is consistent with the defined diffusion coefficient. In the context of MT, the presented model can be interpreted as having two free water (intra‐ and extracellular) and a bound pool. However, to maintain consistency with the remainder of this manuscript and dMRI literature (that does not typically consider MT), we will describe our approach and findings in terms of the intra‐ and extra‐cellular space in dMRI two‐compartment models.

Beside the intrinsic *T*_1_ and *T*_2_ *s* of the free and bound pools, we control the properties of MT via two characteristics: (1) dwell time characterizes the average time an isochromat remains in the bound pool; (2) surface density is the ratio between surface isochromat density on the obstruction and the volume isochromat density in the free pool. In relation to the conventional binary spin‐bath model, surface density multiplied by surface‐to‐volume ratio controls the proportion of isochromats that are bounded, while dwell time and surface density together control the probability of an isochromat getting bounded when it encounters an obstruction and therefore the overall transfer rate between pools. The transfer probability is determined with the additional constraint that dictates the average rates of isochromats joining and leaving the bound pool should be matched. This ensures that even though each isochromat is modeled independently, the relative size of the bound pool and free pool remains consistent. The simulator uniformly distributes the isochromats in the two pools over their corresponding surface/volume during initialization. See the simulator paper [44] for a more detailed description of the implementation.

In this work we only consider the effects of MT on the transverse magnetization (i.e., effective *T*_2_) of the free water. This is different from typical MT experiments, where the focus is on the transfer of longitudinal magnetization. The transfer of both longitudinal and transverse magnetization are modeled in MCMRSimulator [44]. As we only simulate a single repetition with a single excitation pulse, the MT's influence on the longitudinal magnetization does not impact the estimated transverse magnetization. In realistic dMRI sequences these effects on the longitudinal magnetization do affect the diffusion‐weighted signal through their effect on the steady‐state equilibrium longitudinal magnetization reached over multiple repetition times. The use of a single excitation pulse means that the values set for the *T*_1_ in the simulations will not affect the simulated dMRI signal or its relationship with MT/permeability, so we arbitrarily set the *T*_1_ to infinity for both the free and bound pools.

###### 2.1.2.2.2 Parameter Selection

While the intrinsic relaxation constants of the free pool were set to infinity, MT introduces relaxation through the bound pool. Isochromats that transfer from the free pool to the bound pool quickly lose their transverse magnetization, reducing the total transverse magnetization of the whole system. As a result, MT effects can be observed and quantified in the *b* = 0 signal as an effective *T*_2_ relaxation, where a shorter effective *T*_2_ means a stronger contribution of MT. Equation (5) defines the relationship between dwell time, surface density and effective *T*_2_ for cylinders. See Supporting Information 1 for a detailed derivation.

(5) $$T_{2}={\left(\frac{S}{V}\right)}^{-1}\frac{\text{dwell time}}{\text{surface density}}=\frac{r}{2}\frac{\text{dwell time}}{\text{surface density}}$$

We also used the surface‐to‐volume ratio of the intra‐axonal signal to remove the volume fraction dependence as in Equation (3) and keep the MT strength a density‐independent property. Our two‐pool implementation is in line with the conventional binary spin‐bath model, which assumes perfect isochromat mixing in the bound pool. A model that captures the MT between the surface and deeper part of the membrane would be more realistic but would still manifest as an effective *T*_2_ decay and should not introduce MT between the intra‐ and extra‐axonal compartments [56].

Appropriate bound pool parameters were set to achieve effective *T*_2_ *s* at the same order of magnitude as the *T*_2_ of white matter (˜80 ms at 3 T). The dwell time was set to 30 ms as reported by Helms et al. [57] and the surface density was varied to achieve different T_2_ values ranging from 30 to 150 ms. The *T*_2_ of the bound pool (macromolecules) was set to 10 μs based on past literature [58, 59], while the *T*_1_ remained infinite. For the distributed cylinder diameter case, the effective *T*_2_ is dependent on the cylinder radius due to the surface‐to‐volume ratio. Therefore, we varied the surface density to achieve different effective *T*_2_ for a cylinder diameter of 4 μm (the mean diameter of the distribution) and used the same surface density for all the cylinders in the set.

### 2.2 Two‐Compartment Model Fitting

To estimate the cylinder diameter and density from a simulated signal, we fit a two‐compartment model to our data. Specifically, we modeled the diffusion‐weighted signal attenuation as the sum of two compartments corresponding to intra‐axonal and extra‐axonal water, defining:

(6) $$E=f_{i}E_{i}+\left(1-f_{i}\right)E_{e}$$

where *f*_*i*_ is the intra‐axonal signal fraction that quantifies the cylinder density, *E*_*i*_ is the intra‐axonal signal attenuation and *E*_*e*_ is the extra‐axonal signal attenuation. We modeled the extra‐axonal signal as a free diffusion compartment that exhibits Gaussian diffusion and uniform radial diffusivity perpendicular to the cylinder axis. Thus, the extra‐axonal signal is represented by:

(7) $$E_{e}=e^{-bD_{e}}$$

where *b* is the *b*‐value and *D*_*e*_ is the diffusivity of the extra‐axonal space. Note that due to hindrance of the cylinders *D*_*e*_ is not necessarily the intrinsic diffusivity and therefore will need to be estimated from the data.

For the intra‐axonal signal, we used the MCMRSimulator to generate a dictionary of intra‐axonal signals from parallel cylinders with different diameters at a spacing of 0.2 μm. We subsequently constructed a projection from cylinder diameter to the intra‐axonal signal by interpolating between the discrete value pairs in the simulated dictionary. This approach allowed us to model intra‐axonal signals more accurately than existing analytical models based on Gaussian phase approximation [60, 61] (see Supporting Information 2.1). We then used these representations of the intra‐axonal and extra‐axonal signal attenuations to describe the total diffusion attenuation via:

(8) $$E=f_{i}\cdot E_{i}\left(d\right)+\left(1-f_{i}\right)\cdot e^{-bD_{e}}$$

Fitting Equation (8) to a simulated dMRI signal allows us to estimate the cylinder density (i.e., intra‐axonal signal fraction, *f*_*i*_), mean cylinder diameter (*d*), and extra‐axonal diffusivity (*D*_*e*_).

Data analysis was performed with Python (version 3.11). The simulated dictionary was interpolated using the CubicSpline function in SciPy [62, 63, 64]. We used the minimize function from SciPy (Nelder–Mead algorithm) to fit our model to the simulated data and estimate three microstructural parameters: cylinder diameter, density and *D*_*e*_. The optimization was constrained with the following conditions to prevent unrealistic estimates: $d\in \left[0,20\right]\mu m,\;f_{i}\in \left[0,1\right],D_{e}\in \left[0,4\right]\mu m^{2}/\mathrm{ms}$. The intra‐axonal diffusivity was set to 2.3μm^2^/ms, same as the intrinsic diffusivity.

## 3 Results

Relevant code and raw data are available online at https://github.com/zhiyuzheng1769/MT‐and‐permeability‐effect‐on‐two‐compartment‐dMRI‐WM‐model. As defined in Section 2.1.1.1 we present results from simulations using a substrate with uniform cylinder diameters (fixed diameter case) and distributed cylinder diameters (distributed diameter case).

### 3.1 No MT and Permeability

**FIGURE 4:**
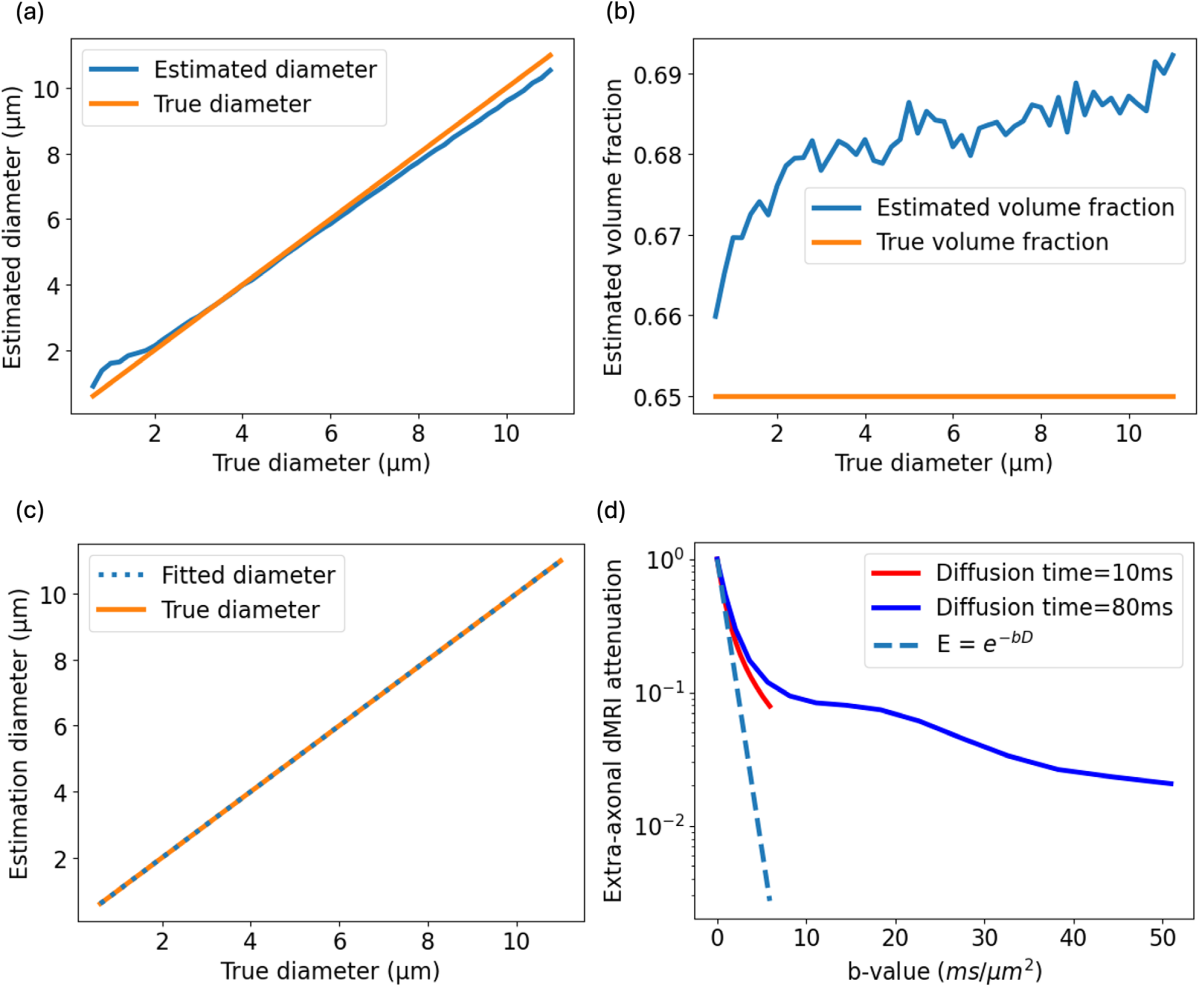
Parameter estimation and simulated signal with no permeability or MT (fixed diameter case). (a) Cylinder diameter and volume fraction estimates without permeability or MT. (c) Cylinder diameter estimates based on a model including only the intra‐axonal signal compartment, and (d) simulated extra‐axonal signal attenuation. The extra‐axonal attenuation is not linear with respect to *b*‐value on the semilog plot, inconsistent with a Gaussian diffusion model ($E=e^{-\mathrm{bD}}$).

Figure 4a,b displays the estimated diameter and volume fraction as a function of the true underlying cylinder diameter for the fixed diameter case. We observed a small bias in the estimates for cylinder diameter and volume fraction as a function of cylinder diameter, with diameter estimate errors within ±1 μm and < 0.05 for the volume fraction estimation. Separating the intra‐axonal and extra‐axonal signals (Figure 4c,d), we observed that while the cylinder diameter can be accurately fitted by our simulation‐based intra‐axonal signal model, the extra‐axonal signal compartment does not exhibit Gaussian diffusion behavior across all investigated diffusion regimes. Further investigation found that including higher order non‐Gaussian diffusion terms (kurtosis or time‐dependent diffusion) in the extra‐axonal signal model did not improve the estimation accuracy (Supporting Information 2.2). To account for the small bias in Figure 4a,b, we used these biased estimates (i.e., from the no MT or permeability case) rather than the ground truth as the baseline for the following sections. This allows us to evaluate on the impact of permeability and MT on parameter estimates.

### 3.2 Permeability

**FIGURE 5:**
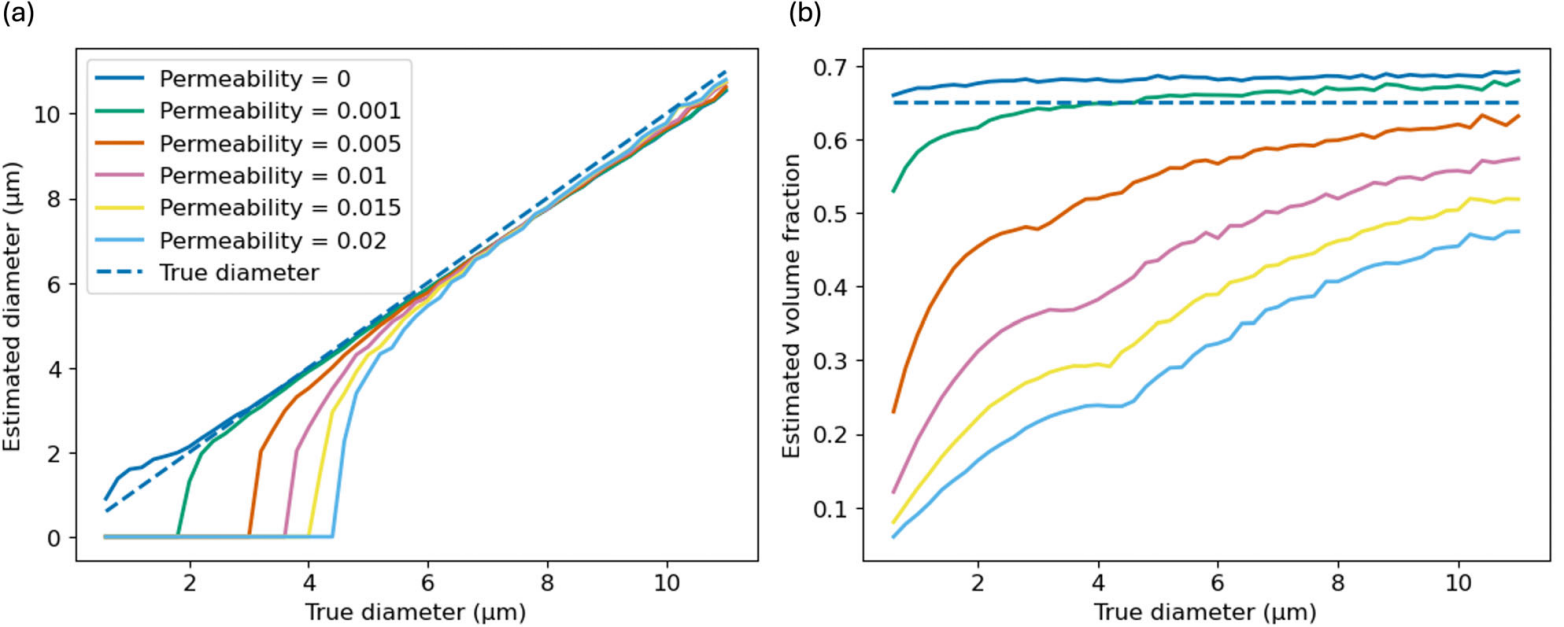
Diameter (a) and volume fraction (b) estimation with semipermeable cylinders, fixed diameter. Permeability caused a reduction in the estimates in both diameter and volume fraction. The diameter estimates additionally exhibit a cutoff behavior, going to zero (the lower bound) when the underlying diameter is below a permeability‐dependent cutoff diameter.

Figure 5 displays the estimated diameter and volume fraction for semipermeable cylinders (fixed diameter case). The results show that introduction of membrane permeability leads to diameter and volume fraction underestimation. For both cylinder diameter and volume fraction estimation, we observe that the bias relative to the no permeability case becomes larger as the true cylinder diameter decreases and as the permeability of the cylinder wall increases. Notably, there exists a cutoff diameter as a function of permeability, below which the signal model estimates zero diameter. Furthermore, we see that a low level of permeability—0.001, corresponding to an exchange time of 508 ms for a 2 μm‐diameter cylinder, is already enough to cause visible underestimation in diameter and volume fraction for small cylinders.

**FIGURE 6:**
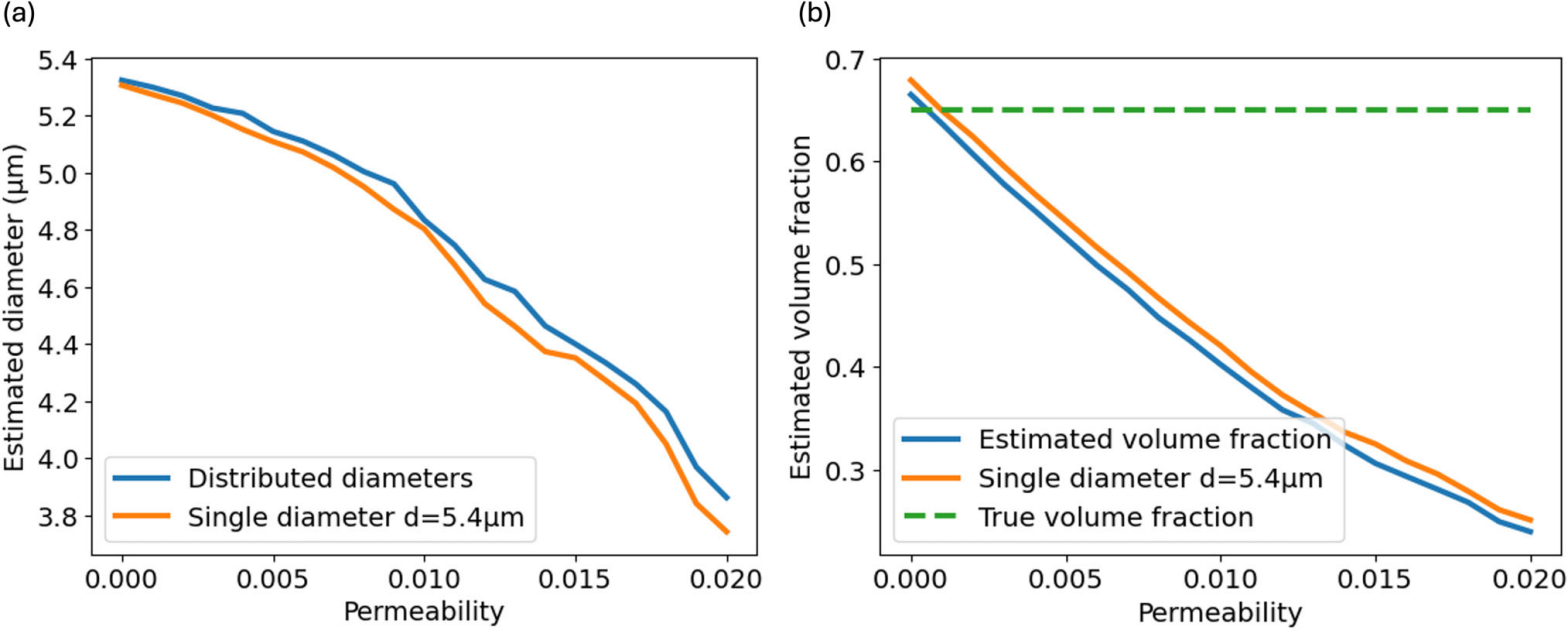
Diameter (a) and volume fraction (b) estimation with semipermeable cylinders, gamma distributed diameter with mean of 4 μm and variance of 1μm^2^. The estimate from a single diameter of 5.4 μm is plotted to show the similarity in the estimate variation between the distributed diameter case and a uniform diameter case.

When the cylinder diameters became Gamma‐distributed, we also observed an underestimation of the cylinder diameter and volume fraction, as shown in Figure 6. The diameter estimation exhibited a similar trend to a single diameter case (orange line in Figure 6a).

### 3.3 Magnetization Transfer

**FIGURE 7:**
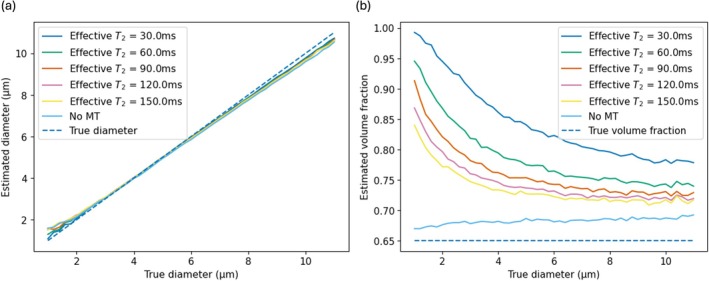
Diameter (a) and volume fraction (b) estimation with cylinders with MT, uniform diameter. MT strength had little effect on the diameter estimation but caused large overestimation of the volume fraction. Note that the effective *T*_2_ is calculated relative to cylinders with a diameter of 2 μm. As the effective *T*_2_ depends on surface‐to‐volume ratio, the actual effective *T*_2_ decreases as the true diameter decreases, which creates the diameter dependence in volume fraction overestimation.

Figure 7 plots the estimated diameter and volume fraction as a function of MT strength represented by different effective *T*_2_ values. The lower the *T*_2_, the stronger the MT effect is. The figure shows that for the fixed diameter case, diameter estimation is broadly independent of MT strength. However, MT led to large overestimations of the volume fraction across all diameters, and the overestimation was larger at smaller axon diameters.

**FIGURE 8:**
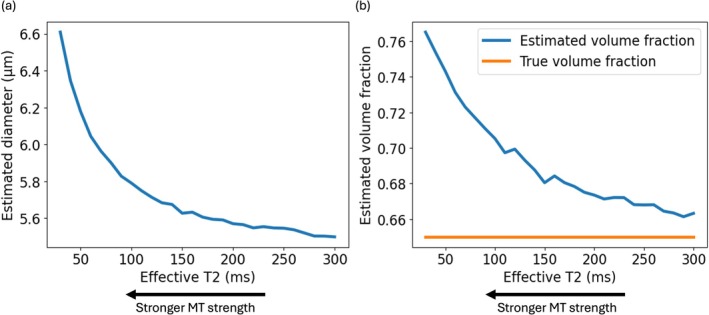
Diameter (a) and volume fraction (b) estimation with cylinders with MT, gamma distributed diameter with mean of 4 μm and variance of 1 μm^2^. MT caused overestimation in both diameter and volume fraction of cylinders. Note the effective *T*_2_ decreases as the MT becomes stronger.

In the distributed case, we observed an overestimation of both cylinder diameter and volume fraction, as shown in Figure 8. The bias increased as MT strength increased.

## 4 Discussion

### 4.1 Permeability Effects

**FIGURE 9:**
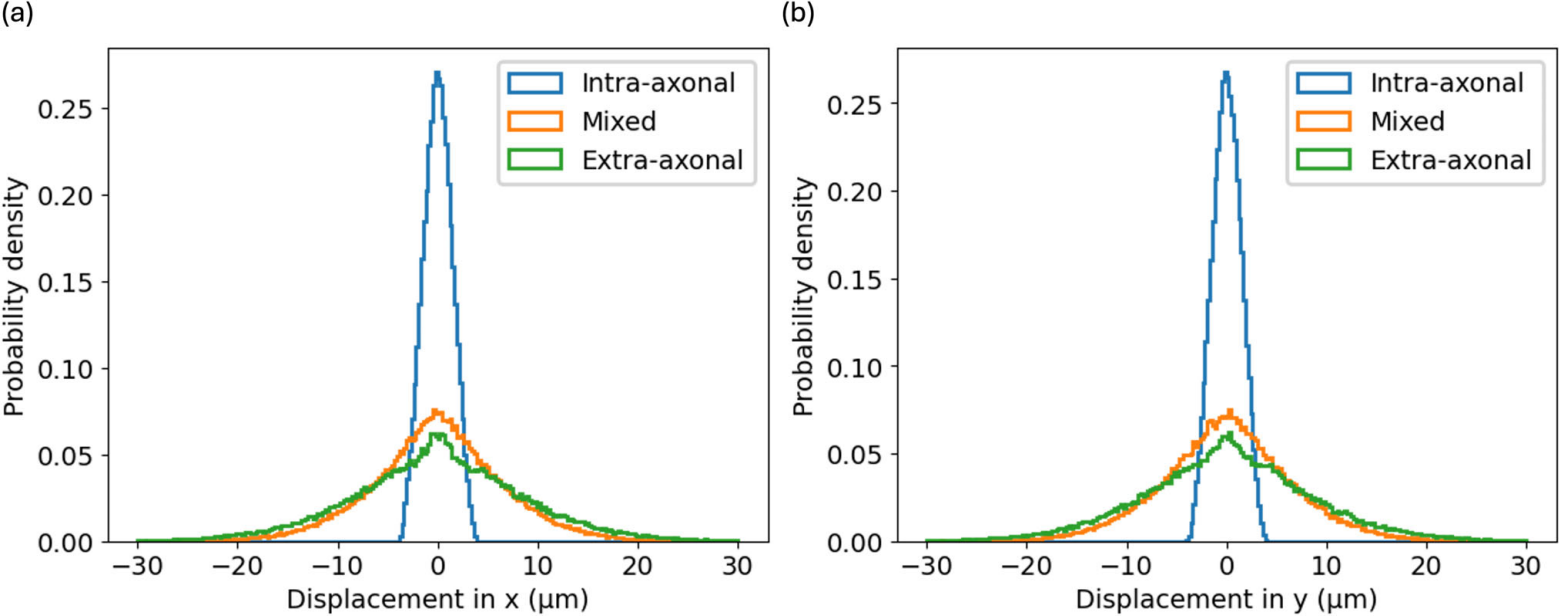
Displacement profile of isochromats along the axes perpendicular to the cylinders. This illustrates isochromats that are within the intra‐axonal or extra‐axonal space throughout the simulation, and isochromats that spend half of the time in intra‐axonal space and half in extra‐axonal space (“mixed”). It can be observed that the isochromats that spend half of the time in each compartment have a displacement profile much closer to the extra‐axonal displacement profile. This leads to an apparent increase in the extra‐axonal signal fraction and underestimation of the axon volume fraction.

The underestimation of the volume fraction observed in Figure 5b has a direct link with permeability. Permeability causes any isochromats that have spent some time in the extra‐axonal space to have a displacement profile closer to an extra‐axonal isochromat than an intra‐axonal isochromat (see Figure 9). This leads to a large intra‐axonal signal reduction under the dMRI encoding and causes the volume fraction to be underestimated in all cases regardless of cylinder diameter. In the fixed diameter case, the underestimation bias is larger for smaller diameters. This is because the macroscopic exchange rate is larger for smaller cylinders for the same permeability value, due to a larger surface‐to‐volume ratio (see Equations (2), (3), (4) and Figure 2b).

The underestimation of the cylinder diameter in semipermeable cylinders (Figure 5a) arises from the construct of the two‐compartment model, which associates time‐dependent non‐Gaussianity in the dMRI signal with the intra‐axonal component (Equation 8). For impermeable cylinders, restricted intra‐axonal diffusion and unrestricted extra‐axonal diffusion lead to increasing differences in apparent diffusivities with diffusion time (Δ), producing positive kurtosis and a positive *dK*/*d*Δ. This effect is diameter‐dependent: small axons show little change in kurtosis because intra‐axonal diffusivity is already near zero at short Δ, whereas larger axons exhibit a stronger and more prolonged increase in kurtosis. However, when cylinder walls are semipermeable, exchange across the boundary increases with Δ, promoting mixing between compartments. This reduces the difference in apparent diffusivities and suppresses the diffusion‐time dependence of kurtosis. As a result, *dK*/*d*Δ decreases with increasing permeability, counteracting the effect of restriction, consistent with previous observations [65, 66].

In the two‐compartment model, the extra‐axonal compartment is Gaussian and time‐independent, and axon density only scales compartmental signal fractions. Consequently, the cylinder diameter is the only parameter governing *dK*/*d*Δ during fitting. Reduced *dK*/*d*Δ caused by permeability is, therefore, interpreted as a smaller diameter, leading to systematic underestimation and, at high permeability, collapse of the estimate to its lower bound, zero (Figure 5a). This effect is more pronounced for small axons, where even weak permeability can fully cancel their small restriction‐induced *dK*/*d*Δ. The same mechanism explains the underestimation observed in the distributed diameter case (Figure 6a) and is consistent with prior reports of restriction‐size underestimation in permeable spherical substrates [67]. See Supporting Information 3 for a more detailed kurtosis analysis.

### 4.2 MT Effects

When considering MT, the overestimation of volume fraction shown in Figures 7b and 8b can be explained by the surface‐to‐volume ratio differences. Specifically, the intra‐axonal compartment has a volume fraction of 0.65, larger than the extra‐axonal compartment. Because the intra‐ and extra‐axonal compartments share the same surface area (the cylinder walls), the extra‐axonal compartment has a larger surface‐to‐volume ratio. Since we modeled MT as a surface process, isochromats in the compartment that has a larger surface‐to‐volume ratio will interact with the cylinder walls more frequently and relax faster. Thus, the extra‐axonal signal has a shorter effective *T*_2_ and contributes a smaller fraction of the total signal. Therefore, the relative fraction of signals that comes from the intra‐axonal compartment increases with MT strength and makes the model predict a larger volume fraction estimate. Past literature has also reported that the intra‐axonal compartment in white matter tends to have a larger effective *T*_2_ than the extra‐axonal compartment [68, 69]. We note further that in our simulation the cylinder walls were infinitesimally thin which led to the two compartments having the same surface area. In reality, the axon membrane has finite thickness and myelin sheath thickness will add further to the total thickness. This will make the extra‐axonal compartment have a larger surface area than the intra‐axonal compartment and amplify the surface‐to‐volume ratio difference between the two compartments. So, we expect a greater volume fraction overestimation caused by MT in practice. In addition, this surface‐to‐volume ratio argument could also explain the extremely short *T*_2_ of myelin water: myelin sheath's spiral configuration leads to a large surface‐to‐volume ratio and therefore very short MT‐induced effective T_2_.

**FIGURE 10:**
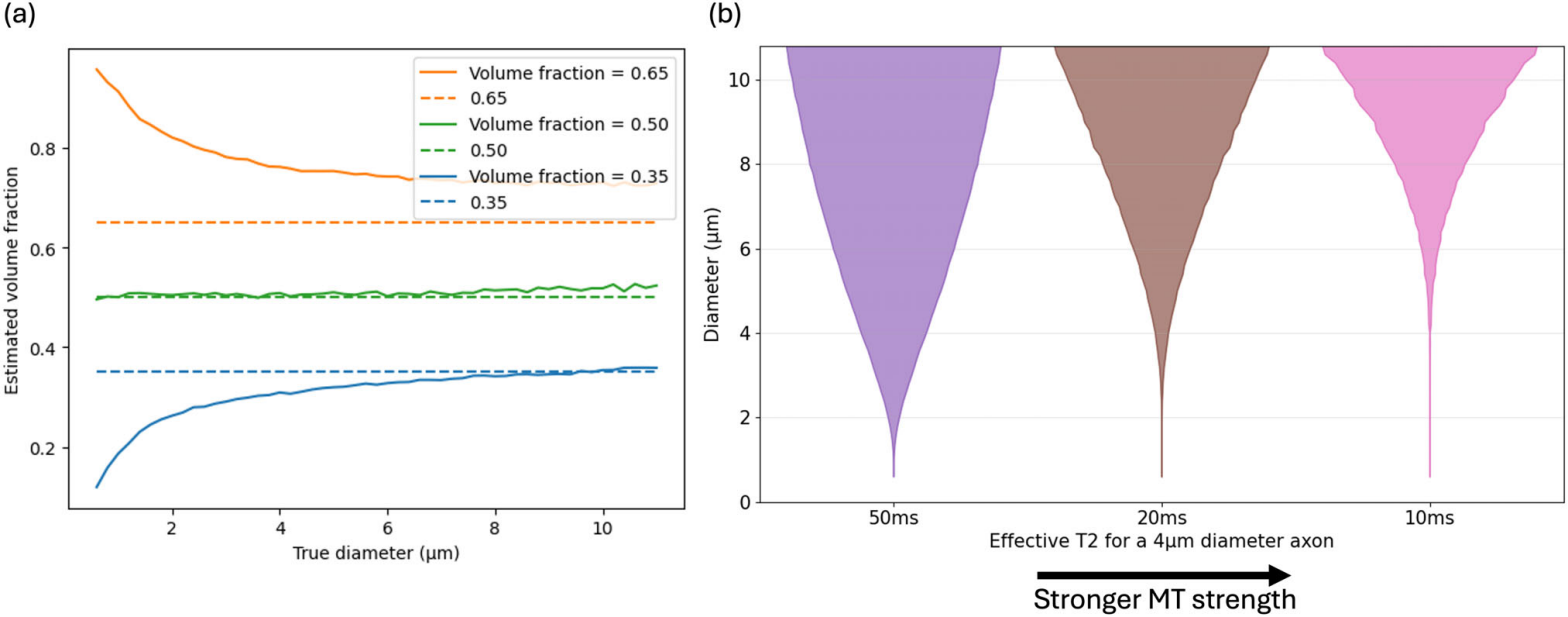
MT results interpretations: (a) volume fraction estimates with different underlying volume fractions (0.35, 0.5, 0.65, marked by the dashed lines). All three substrates had the same MT strength (effective *T*_2_ = 90 ms). It can be observed that an underlying volume fraction greater than 0.5 (i.e., 0.65) causes overestimations of volume fraction while an underlying volume fraction smaller than 0.5 (i.e., 0.35) causes underestimations of volume fraction. (b) Relative signal levels (the width of the violin plot) for axons of different diameters when incorporating magnetization transfer that achieves effective *T*_2_ *s* of 10, 20 and 50 ms for a 4 μm‐diameter axon. As MT effect strengthens (effective *T*_2_ decreases), there is a reduced relative signal contribution from smaller diameter axons comparing to larger ones.

Further investigations confirmed the volume fraction estimation bias was related to the underlying true volume fraction. When we altered the true volume fraction to 0.5 and 0.35, the overestimation of volume fraction became much smaller (0.5) or underestimated (0.35), the opposite effect to the 0.65 volume fraction case (see Figure 10a).

As for the diameter dependence of the overestimation observed in Figures 7b and 8b, Equation (5) shows that effective *T*_2_ of the intra‐axonal compartment is inversely proportional to the surface‐to‐volume ratio and proportional to axon radius. Similarly, the extra‐axonal compartment's effective *T*_2_ is also proportional to axon radius but has a smaller coefficient as the surface‐to‐volume ratio is larger. The ratio of signal contribution from the two compartments is

(9) $$\frac{S_{\text{intra}}}{S_{\text{extra}}}=\frac{{S_{\text{intra}}}_{0}}{{S_{\text{extra}}}_{0}}\frac{e^{-\frac{\mathrm{TE}}{{T_{2}}_{\text{intra}}}}}{e^{-\frac{\mathrm{TE}}{{T_{2}}_{\text{extra}}}}}=\frac{{S_{\text{intra}}}_{0}}{{S_{\text{extra}}}_{0}}e^{-\frac{\mathrm{TE}}{A_{\text{intra}}\cdot r}+\frac{\mathrm{TE}}{A_{\text{extra}}\cdot r}}=\frac{{S_{\text{intra}}}_{0}}{{S_{\text{extra}}}_{0}}e^{\frac{\mathrm{TE}}{r}\left(\frac{1}{A_{\text{extra}}}-\frac{1}{A_{\text{intra}}}\right)}$$

where ${S_{\text{intra}}}_{0}$ and ${S_{\text{extra}}}_{0}$ are initial signal levels of the two compartments and *A*_intra_ and *A*_extra_ are the proportionality coefficients that relate the axon radius to the effective *T*_2_ of respective compartments. This ratio determines the volume fraction estimate we get and it has direct dependence on axon radius. When *A*_extra_ < *A*_intra_ (i.e., ${T_{2}}_{\text{extra}}<{T_{2}}_{\text{intra}}$, which is our case), the ratio increases as the radius (diameter) decreases, which matches the trends in Figures 7b and 8b.

As for the diameter estimation, since MT does not affect the time‐dependence of the kurtosis as permeability does, this explains the minimal change in diameter estimate with MT in fixed diameter cases. MT still affects the kurtosis by altering the relative signal contributions from different compartments, but this is captured in the change in the estimated density already.

When the axon diameter became Gamma‐distributed, we additionally have to consider the diameter dependence of the macroscopic MT effect. The MT strength is controlled by two diameter‐independent parameters—surface density and dwell time (see Section 2.1.2), with the effective *T*_2_ dependent on the axon diameter. Since we set the same surface density and dwell time for all cylinders, Equation (5) predicts that in axons smaller diameter leads to a shorter effective *T*_2_ and larger diameter leads to a longer effective *T*_2_. This matches an experimental observation reported previously [70]. This prediction was verified when we simulated the signal level for a wide range of axon diameters with the same surface density and dwell time (that for a 4 μm‐diameter axon would lead to effective *T*_2_ of 50, 20, and 10 ms), as shown in Figure 10b. Specifically, we observe a biased weighting in the total signal toward the larger axons as their signal decays more slowly than the smaller axons and the large axon dominance gets stronger as MT effect becomes stronger (effective *T*_2_ decreases). Therefore, during parameter estimation the axon diameter was overestimated as shown in Figure 8a.

### 4.3 Model Selection

In this project, we used a simple, two‐compartment parallel cylinder model (i.e., AxCaliber). The motivation was to use a model with well‐established properties and limited degeneracies. While we focused on the impact of MT/permeability on axon radius and density estimates, the proposed approach could be extended to microstructural models that incorporate more sophisticated features (e.g., fiber dispersion in ActiveAx) to understand how these estimates are similarly impacted. Furthermore, we have performed preliminary experiments that showed the observation in the results section extends to a reduced acquisition protocol with only the long diffusion time shells (Supporting Information 4). This suggests that our findings may also apply to other acquisition protocols with different sequence parameters despite the change in MT/permeability sensitivity.

Our initial investigation without MT and permeability effects observed a discrepancy between the estimated model parameters and the true underlying parameters from Monte‐Carlo simulations (Figure 4a,b). Further investigation identified that this discrepancy arose from the extracellular space, where the signal model's assumption of free‐Gaussian diffusion was not valid across the diffusion regimes investigated in this study (Figure 4d). However, limited improvement was observed when fitting a time‐dependent model [47] to the extracellular space (Supporting Information 2.2). Typically, the radius bias was a fraction of a micron over the investigated parameter space, with the volume fraction bias < 0.05.

### 4.4 Impact

Given these results and interpretations, we argue that MT and permeability can indeed have a non‐negligible effect on the dMRI signal. We observed biases in estimated microstructural features (e.g., axon diameter) from the affected dMRI signal using a two‐compartment model when MT and permeability are present. More specifically, we noticed that the bias induced by MT and permeability increases as the axon diameter decreases (Figures 5 and 7b). This is because smaller axons have a larger surface‐to‐volume ratio, which leads to a shorter effective *T*_2_ with MT and a shorter exchange time with permeability. Furthermore, smaller axons are more likely to be unmyelinated, which leads to even faster exchange through permeability and shorter exchange time. Taken together, MT and permeability's presence biases dMRI signal further toward the signals generated by larger axons on top of the previously reported geometry effects [18, 71, 72].

Noticeably, in Figure 5, we demonstrate that a small amount of permeability is enough to make a two‐compartment model insensitive to axon diameters below 2 μm: Specifically, a permeability of 0.001, which translates to an exchange time of ˜500 ms for axon diameter of 2 μm, is enough to cause the estimated diameter to go to zero for axons smaller than 2 μm. Many axons in white matter have diameters below 2 μm [1, 29, 47, 73] and some studies have suggested the white matter exchange time ranges from 150 to 900 ms [74, 75, 76]. In addition, for unmyelinated neurons the exchange time measured in gray matter was also shorter than 500 ms [18, 75, 77]. We also demonstrated that MT could bias the axon density estimation through the surface‐to‐volume ratio difference induced by the underlying volume fractions. The effective *T*_2_ levels needed to induce a visible bias are comparable to realistic white matter *T*_2_ values. Our results, therefore, suggest realistic and biologically relevant MT strength and permeability values can bias dMRI estimates. Thus, dMRI modeling methods that incorporated permeability and multicompartmental relaxation should be considered to produce more accurate estimation of microstructural parameters. No white matter model currently incorporates non‐Gaussian diffusion, permeability and MT.

We performed our investigations using Monte‐Carlo simulations, observing quantitative effects of MT and permeability on dMRI microstructural estimates. Monte‐Carlo simulations allowed us to characterize these effects without having to define complicated analytical equations. Further investigations of more complex geometries are also possible without extra analytical derivations. We have made the code and example data available online at https://github.com/zhiyuzheng1769/MT‐and‐permeability‐effect‐on‐two‐compartment‐dMRI‐WM‐model/tree/main for readers who may be interested in exploring these findings further.

In our investigations we chose a two‐compartment dMRI signal model which models the intra‐axonal signal using a parallel cylinder model. As the effect of MT and permeability is only coupled with the surface‐to‐volume ratio and not the geometry type, our observations should generalize to other dMRI signal models using different geometries (e.g., spheres). In addition, many dMRI white matter signal models share the same two‐compartment framework with the one we used and do not account for MT or permeability. Therefore, our findings should also generalize to a wider group of axon diameter mapping methods and substrates.

## 5 Conclusion

We used Monte‐Carlo simulations to investigate the effect of MT and membrane permeability on the dMRI signal. Using parallel‐cylinder substrates, both MT and permeability caused observable changes in dMRI signal and biased microstructural estimates based on a two‐compartment model. The changes showed similar trends in both fixed and distributed diameter cases, and are expected to be generalizable to other white matter dMRI signal models and other substrates. Findings demonstrate that Monte‐Carlo simulations can provide insights into how MT and permeability can impact dMRI signals, complementing analytical approaches.

## Funding

This work was supported by NIHR Oxford Health Biomedical Research Centre (NIHR203316), University of Oxford, China Scholarship Council, Wellcome Trust (203139/A/16/Z, 203139/Z/16/Z, 215573/Z/19/Z, 222829/Z/21/Z, and 224573/Z/21/Z).

## Supporting information


**Figure S1:** Intra‐axonal signal model selection.
**Figure S2:** Extra‐axonal signal model selection.
**Figure S3:** Kurtosis variation with diffusion time, cylinder diameter, and permeability.
**Figure S4:** Short diffusion time sequences' effect on permeability‐biased estimation results.
**Figure S5:** Short diffusion time sequences' effect on MT‐biased estimation results.

## Data Availability

All the scripts and raw data that are used to obtain the following results are available online at https://github.com/zhiyuzheng1769/MT‐and‐permeability‐effect‐on‐two‐compartment‐dMRI‐WM‐model. The SHA‐1 hash for the version used is 96b6466de1e743e828d3b3f7aad31a3d8a6 ac1c3.
