# Supplementary material for: Investigating the Sensitivity of the Diffusion MRI Signal to Magnetization Transfer and Permeability via Monte‐Carlo Simulations
Source: Magn Reson Med. 2026 Apr 12;96(2):960–74. doi: 10.1002/mrm.70378 (PMC13269245; doi:10.1002/mrm.70378)
Supplement: Supplementary file 1 — Figure S1: Intra‐axonal signal model selection. Figure S2: Extra‐axonal signal model selection. Figure S3: Kurtosis variation with diffusion time, cylinder diameter, and permeability. Figure S4: Short diffusion time sequences' effect on permeability‐biased estimation results. Figure S5: Short diffusion time sequences' effect on MT‐biased estimation results. [file MRM-96-960-s001.pdf]

Supporting information

1 Mathematical relationship between simulator MT variables and effective T_2

To convert between the simulator MT variables (dwell time and surface density) and the effective T_2, we need to account for the surface-to-volume ratio (S/V), which is the surface area of the geometry (S) divided by the volume of interest (V). The effective T_2 can then be derived from the rate of isochromats leaving the free pool (r_{out}) as the isochromats effectively lose their transverse magnetization instantly when they transfer from the free to the bound pool:

$$\frac{dE}{dt} = -\frac{r_{out}}{N_{free}}E$$

$$E = e^{-\frac{r_{out}}{N_{free}}t} = e^{-\frac{t}{T_2}}$$

$$T_2 = \frac{N_{free}}{r_{out}} \tag{S1}$$

where E is the signal attenuation, t is time, and N_{free} is the number of isochromats in the free pool. The rate of leaving is the same as the rate of entering the free pool (leaving the bound pool) at equilibrium. The rate of entering (r_{in}) is defined as:

$$r_{in} = \frac{N_{bound}}{dwell\ time} \tag{S2}$$

where N_{bound} is the number of isochromats in the bound pool and dwell time is the average time it takes for a bounded isochromat to be released back to free pool. As $r_{in} = r_{out}$, we can combine Eq.S1 and Eq.S2:

$$T_2 = \frac{N_{free}}{N_{bound}} \times (dwell\ time) \tag{S3}$$

This equation can also be obtained using the conventional binary spin bath model by having a bound pool with infinitely short T_2 and therefore negligible transverse signal. Then the total signal attenuation is just the free pool's signal attenuation. Because the intrinsic T_2 of the free pool was ignored and set to infinity, the only signal loss in the free pool is the isochromats being transferred to the bound pool, which leads to Eq.S1.

Recall that the surface density is the ratio between surface isochromat density on the

obstruction and the volume isochromat density in the free pool. Therefore:

$$surface\ density = \frac{\frac{N_{bound}}{S}}{\frac{N_{free}}{V}} = \frac{N_{bound}}{N_{free}}\frac{V}{S}$$

$$T_2 = \frac{V}{S}\frac{1}{surface\ density} \times (dwell\ time) = \left(\frac{S}{V}\right)^{-1}\frac{dwell\ time}{surface\ density} \qquad (S4)$$

For cylinders:

$$\frac{S}{V} = \frac{2\pi r l}{\pi r^2 l} = \frac{2}{r} \qquad (S5)$$

where r is the radius, l is the length. Therefore, we can use Eq.S4 (Eq.5 in 2.1.2.2) to get effective T_2 for the intra-axonal water directly from the dwell time and surface density values.

2 Selection of compartment models in the two-compartment model

2.1 Evaluating analytical and simulation-based models for the intra-axonal signal

To accurately model the intra-axonal signal, we used MCMRSimulator to generate a dictionary of intra-axonal signals from parallel cylinders with different diameters at a spacing of 0.2μm. We then constructed a projection from cylinder diameter to the intra-axonal signal by interpolating between the discrete value pairs in the simulated dictionary. This allowed us to leverage the Monte-Carlo simulation which provides the ground truth for our modelling and make fewer assumptions versus existing analytical models (e.g. Callaghan[1], Van Gelderen[2]) which are based on the Gaussian Phase Approximation. As shown in Figure S1, breakdown of these assumptions in restricted substrates leads to deviation in the estimated cylinder diameter, while our interpolated dictionary approach gave accurate estimates.

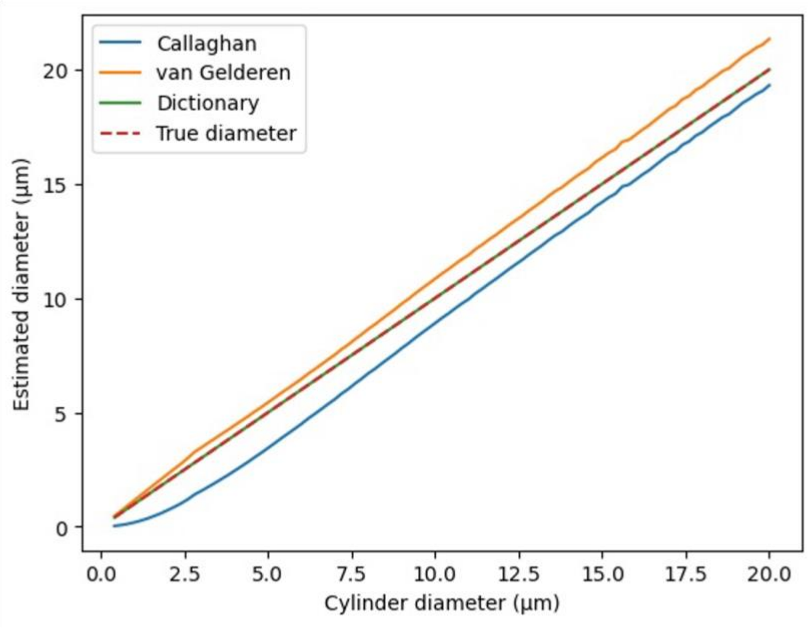

Figure S1: **Intra-axonal signal model selection**: Figure S1 shows the diameter estimate using the intra-axonal signal with different signal models. Both Callaghan and van Gelderen models' (which are based on the Gaussian phase approximation) estimations deviated from the ground truth generated by Monte-Carlo simulation.

2.2 Evaluating time dependence and kurtosis models to characterize the extra-axonal signal

To effectively characterize the extra-axonal signal, we performed comparisons using a (1) Gaussian, (2) time-dependent and (3) kurtosis model. Specifically, the Gaussian model was implemented as:

$$E_e = e^{-bD_e}$$

where E_e is the extra-axonal signal attenuation, b is the b-value of the applied sequence, D_e is the diffusivity of the extra-axonal space.

The time dependent model was implemented as:

$$E_e = e^{-b\left(D_\infty + A\left(\frac{\ln\left(\frac{\Delta}{\delta}\right)+\frac{3}{2}}{\Delta-\frac{\delta}{3}}\right)\right)}$$

where D_∞ is the bulk diffusivity, A is a characteristic coefficient, Δ is the diffusion time, δ is the gradient duration. This implementation was initially proposed by De Santis et al.[3].

And the kurtosis model was implemented as:

$$E_e = e^{-bD_e+\frac{1}{6}b^2D_e^2K}$$

where K is the kurtosis.

As shown in Figure S2, we found that the three models produced similar trends across the investigated diameter and (intra-axonal) volume fraction regimes. Specifically, the Gaussian and kurtosis models yielded almost identical results. The time-dependent model yielded some improvement in diameter estimation in small diameter regimes, and volume fraction in high diameter regimes but its estimates remained noisy.

Given the small differences between the three extra-axonal models and the additional parameters required for the time-dependent or kurtosis model, we proceeded with the Gaussian diffusion model. This approach is consistent with many existing two-compartment models of white matter and facilitates characterization of the extra-cellular signal compartment.

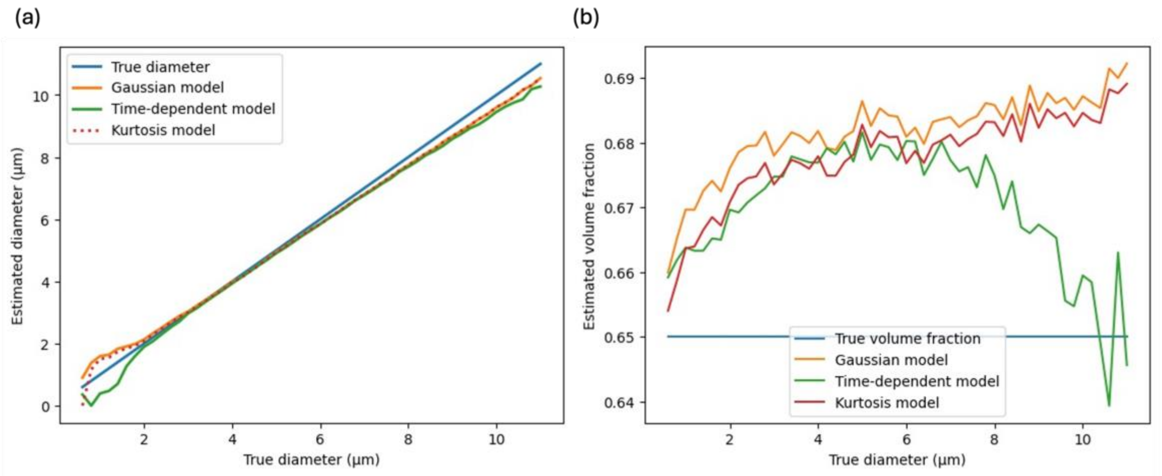

Figure S2: **Extra-axonal signal model selection**: Figures S2a&S2b show Axon diameter and volume fraction estimates without permeability and MT using different extra-axonal signal models in the fixed diameter case. The performance of the kurtosis model is very similar to the Gaussian model. The time-dependent model outperformed the Gaussian model in the volume fraction estimation of large cylinders and diameter estimation of small cylinders.

3 Kurtosis quantification for semi-permeable cylinders

Our two-compartment model associates any time-dependent non-Gaussianity in the dMRI signal with the intra-axonal component and use it to estimate the cylinder diameter. To understand why we observed an underestimation of cylinder diameter in the semi-permeable cylinders, we quantified the non-Gaussianity in the simulated diffusion-weighted signal by estimating its kurtosis. Kurtosis K is defined in a second order correction for the Gaussian diffusion signal model to capture the non-Gaussianity:

$$\frac{S}{S_0} = e^{-bD+\frac{1}{6}b^2D^2K}$$

Where S is the diffusion-weighted signal, S_0 is the non-diffusion-weighted signal, b is the b value, D is the apparent diffusivity. To estimate the kurtosis, we fitted a quadratic function of the b-value to the natural logarithm of the diffusion-weighted signal attenuation $\frac{S}{S_0}$. The coefficient of the second order term is then $\frac{1}{6}D^2K$ and D can be directly obtained as the coefficient of the first order term.

Figure S3a displays the kurtosis estimates for impermeable cylinders at different diffusion times (Δ) as a function of cylinder diameter. As the diameter increases, we observe an increase in the time-derivative of the kurtosis dK/dΔ arising from the restriction size effect: at a diameter around 2μm, kurtosis only increases less than 0.1 as the diffusion time increases from 10 to 40ms, whereas the same increase in diffusion time caused an increase of 0.4 in kurtosis when the diameter is 8μm.

Figure S3b displays the kurtosis estimates at different diffusion times as a function of permeability for a fixed cylinder diameter (4μm). As the permeability increases, dK/dΔ starts decreasing, displaying the opposite trend to Figure S3a. Specifically, the kurtosis at shorter diffusion times is smaller than the kurtosis at longer diffusion times for low permeability, with the opposite trend at high permeability. Taken together, when incorporating permeability, the relationship between diffusion time and kurtosis amplitude becomes more reflective of smaller diameters in the impermeable cylinder case (Figure S3a), leading to the underestimation of diameter when using a two-compartment model.

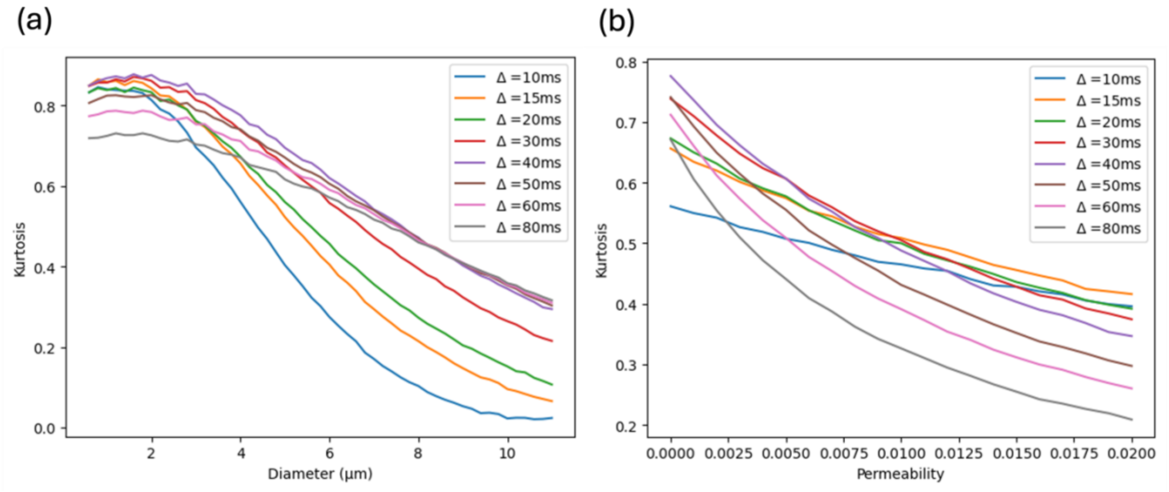

Figure S3: **Kurtosis variation with diffusion time, cylinder diameter and permeability** Figure S3a shows the kurtosis at different diffusion times across different diameter with impermeable cylinders and Figure S3b shows the kurtosis at different diffusion times across different permeability for cylinders with a fixed diameter of 4µm.

4 Preliminary study on the influence of sequence parameter choice on estimation results

In this work we chose the simulated sequence parameters from the widely-adopted AxCaliber protocol and the two compartment model it used to estimate axon diameters from dMRI data as it has well-established properties and limited degeneracies. Naturally the acquisition protocol's sensitivity to MT/permeability changes if the sequence parameters were altered. To assess whether shorter diffusion time sequences in the acquisition are less sensitive to MT/permeability, we investigated the fitting results for the fixed diameter case using only the signals from sequences with larger diffusion times (30-80ms) and compared them with the fit using all sequences (10-80ms). As shown in Figures S4&S5 below, we found that fitting with only larger diffusion time sequences actually reduced the axon diameter underestimation caused by permeability but didn't affect the MT-related biases in axon density and diameter estimates. This suggests the shorter diffusion time sequences may be more sensitive to permeability.

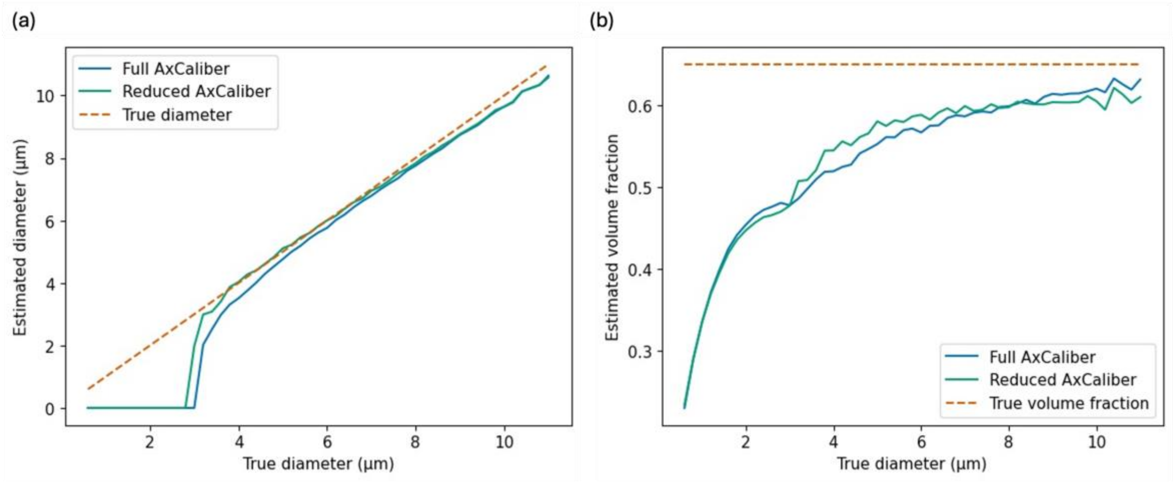

Figure S4: Short diffusion time sequences' effect on permeability-biased estimation results: We constructed the two-compartment model using just the signals from sequences with diffusion time longer or equal to 30ms ("Reduced AxCaliber" case) and fit it to the simulated data. In this case the data was also truncated so only the signals from diffusion time ≥ 30ms are used for fitting. Here we estimated (a) axon diameter and (b) volume fraction the simulated signals with permeability of 0.01 and compared with the original fit in the result section which used all simulated signals from the AxCaliber acquisition ("full AxCaliber" case). The exclusion of shorter diffusion time signals (10-20ms) reduced the underestimation of axon diameter but had a varying effect on the volume fraction estimation as the underlying diameter changes. The other permeability cases in the simulated range (0.001-0.02) showed similar trends.

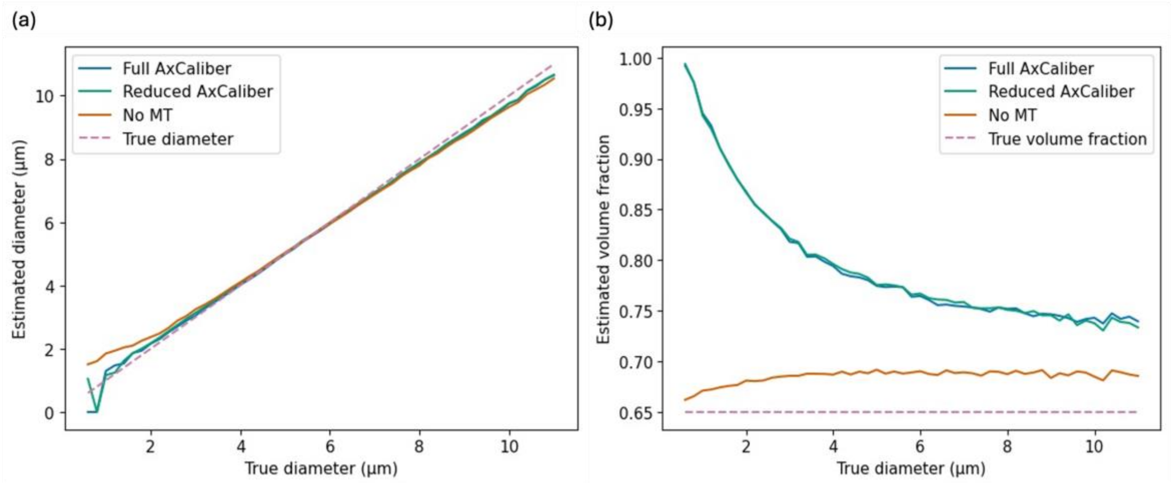

Figure S5: Short diffusion time sequences' effect on MT-biased estimation results: We constructed the two-compartment model using just the signals from sequences with

diffusion time longer or equal to 30ms (“Reduced AxCaliber” case) and fit it to the simulated data. In this case the data was also truncated so only the signals from diffusion time ≥ 30ms are used for fitting. Here we estimated (a) axon diameter and (b) volume fraction the simulated signals with MT-induced effective T_2 of 60ms and compared with the original fit in the result section which used all simulated signals from the AxCaliber acquisition (“full AxCaliber” case). In both of them the estimates in the reduced AxCaliber case were close to those in the full AxCaliber case, suggesting that the short diffusion time sequences are not very sensitive to MT effects.

References

1. Callaghan PT. Pulsed-Gradient Spin-Echo NMR for Planar, Cylindrical, and Spherical Pores under Conditions of Wall Relaxation. *Journal of Magnetic Resonance, Series A*. 1995;113(1):53-59. doi:10.1006/jmra.1995.1055

2. Vangelderen P, Despres D, Vanzijl PCM, Moonen CTW. Evaluation of Restricted Diffusion in Cylinders. Phosphocreatine in Rabbit Leg Muscle. *Journal of Magnetic Resonance, Series B*. 1994;103(3):255-260. doi:10.1006/jmrb.1994.1038

3. De Santis S, Jones DK, Roebroeck A. Including diffusion time dependence in the extra-axonal space improves in vivo estimates of axonal diameter and density in human white matter. *NeuroImage*. 2016;130:91-103. doi:10.1016/j.neuroimage.2016.01.047
